# Stage II Medullary Carcinoma of the Colon: A Surgery Case Report

**DOI:** 10.7759/cureus.50674

**Published:** 2023-12-17

**Authors:** Patrick D Plummer, Benjamin Yglesias, Pablo Giuseppucci

**Affiliations:** 1 Department of Surgery, Trumbull Regional Medical Center, Warren, USA

**Keywords:** laparoscopic colon resection, high risk intervention, adjuvant chemotheapy, stage ii colon cancer, medullary carcinoma of the colon

## Abstract

Medullary carcinoma (MC) is a rare subtype of colorectal cancer, which presents with poorly differentiated histology and is often confused with conventional adenocarcinoma of the colon. While this form of colorectal cancer is rare, it often does not meet the high-risk criteria to qualify for adjuvant chemotherapy even with a favorable prognosis. Diagnosis of MC is a proven difficulty because of the lack of immunohistochemical stains on pathology seen in adenocarcinoma of the colon. Unlike adenocarcinoma of the colon, distant metastasis is rare. Patients diagnosed with MC have one- and two-year survival rates of 93% and 74%, respectively. The patient was a 75-year-old female diagnosed with MC of the sigmoid colon and a large uterine fibroid. In this case report, we discuss the high-risk indications of colorectal cancer and the recommended treatment of patients with stage II MC of the colon.

## Introduction

Medullary carcinoma (MC) of the colon is a rare subtype of colon cancer. The subtypes include poorly differentiated MC (72%) and undifferentiated MC (22%) [1,2]. Generally, MC has a better prognosis than conventional poorly differentiated colonic adenocarcinoma (PDC) [3]. The incidence of MC is extremely rare accounting for only 0.03% of all colorectal carcinomas (CRC) [2]. MC is more prevalent in middle-aged women. In more than 54% of cases, MC is located on the right side of the colon and often presents as an advanced stage of CRC, commonly stage II [2]. Unlike more prevalent forms of CRC, MC is associated with a 10% chance of metastasis [2]. The distinguishable morphology of this cancer is seen in the histology. While most forms of PDC will have pleomorphic glandular appearing cells, MC reveals small- to medium-sized cells with dark nuclei arranged in the form of nests or trabeculae.

Commonly, MC is associated with defective mismatch repair proteins (MMR) [1]. Molecular studies have shown that MC exhibits the absence of hMLH-1 protein by immunohistochemistry [2]. Also, the majority of MC exhibits variable expression of p53 alleles and widespread mutation of microsatellite DNA [3]. In addition, MC is known to be associated with Lynch syndrome (MLH-1) [1,3]. In a study conducted by Winn et al., three immunohistochemical markers commonly seen in MC and PDC were identified [3]. The markers included MLH-1, CDX2, and calretinin, which showed a significant difference in staining with a p-value < 0.05 [3]. In MC, MLH-1 and CDX2 were positive (21% and 19%, respectively) [3]. The most interesting difference was the comparison of calretinin. In MC, calretinin was positive at 73% as opposed to 12% in conventional PDC with a p-value < 0.0001 [3].

Typically, the preferred treatment of non-metastatic colon cancer is composed of a total or partial colectomy and possible adjuvant chemotherapy [4]. In numerous studies, adjuvant chemotherapy (ACT) in patients with stage III or high-risk stage II CRC has proven to improve survival outcomes [4]. In a randomized controlled trial conducted by André et al., the addition of oxaliplatin to the regimen of fluorouracil and leucovorin improves the adjuvant treatment of CRC [5,6].

## Case presentation

**Figure 1:**
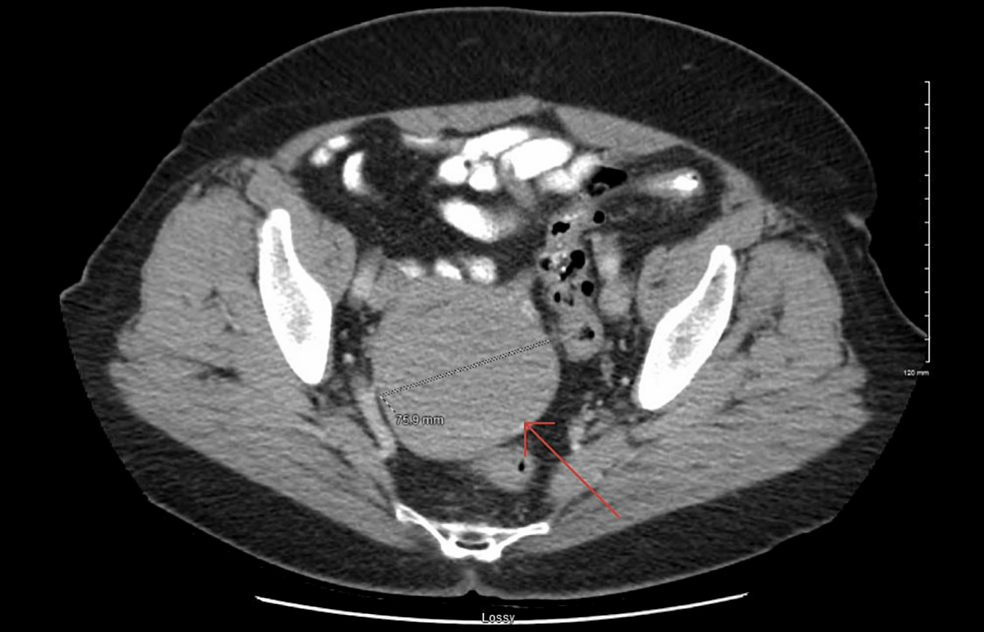
Axial CT scan of the abdomen/pelvis shows a large uterine fibroid (red arrow)

**Figure 2:**
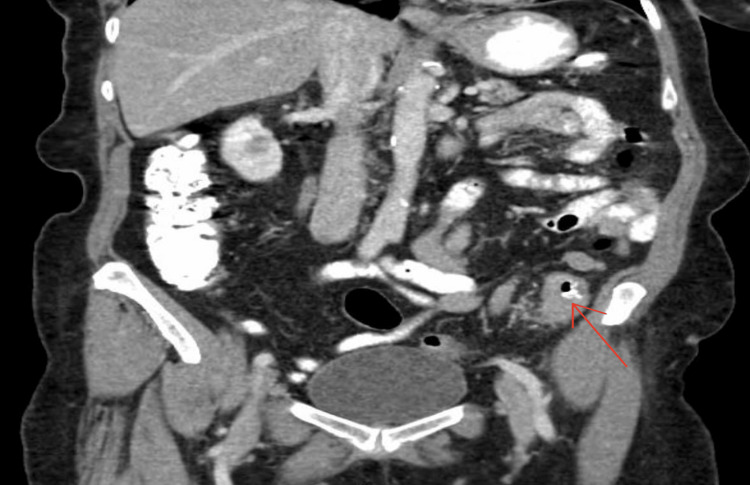
Coronal CT scan of the abdomen shows a thickened segment of the large bowel (red arrow)

The patient is a 75-year-old female with a past medical history of coronary stent placement (on Plavix and aspirin), hypothyroidism, type II diabetes, large uterine fibroid, four to five months of rectal bleeding, and an ulcerated mass in the proximal sigmoid colon found on recent colonoscopy. Biopsies of the colonic mass were believed to be invasive adenocarcinoma of the colon with moderate to poor differentiation. The patient’s CT imaging was negative for metastasis and only demonstrated enlarged uterine fibroid (Figures 1, 2). The patient denied any shortness of breath, nausea/vomiting, palpitations, dizziness, fever, chills, constipation, or diarrhea. Treatment options were discussed with the patient, and she opted for surgical management.

The patient reported to the hospital for an elective robotic laparoscopic total hysterectomy, bilateral salpingo-oophorectomy, and sigmoid colectomy with primary anastomosis. The gynecology team first performed the total hysterectomy and bilateral salpingo-oophorectomy. Next, bilateral ureteral stents were placed through cystoscopy. We then proceeded with our sigmoidectomy. The medial to lateral dissection was started 2 cm distal from the takeoff of the inferior mesenteric artery (IMA) and continued proximally to splenic flexure. Both the descending colon and rectum were dissected circumferentially and transected with a stapler to remove the sigmoid colon. We then proceeded with a primary colorectal anastomosis by using an end-to-end anastomosis (EEA) stapler. The specimen was sent to pathology for further evaluation. After the procedure, the patient had a stable postoperative course. The patient had a return of bowel function on day 3, and her diet was advanced as tolerated. She was discharged in stable condition with no immediate complications. She was instructed to follow up with her oncologist after the surgery to possibly start adjuvant chemotherapy if she was deemed a good candidate. The patient’s case was discussed at a multidisciplinary committee and was not recommended for adjuvant chemotherapy because her stage IIA colon cancer did not have high-risk features.

**Figure 3:**
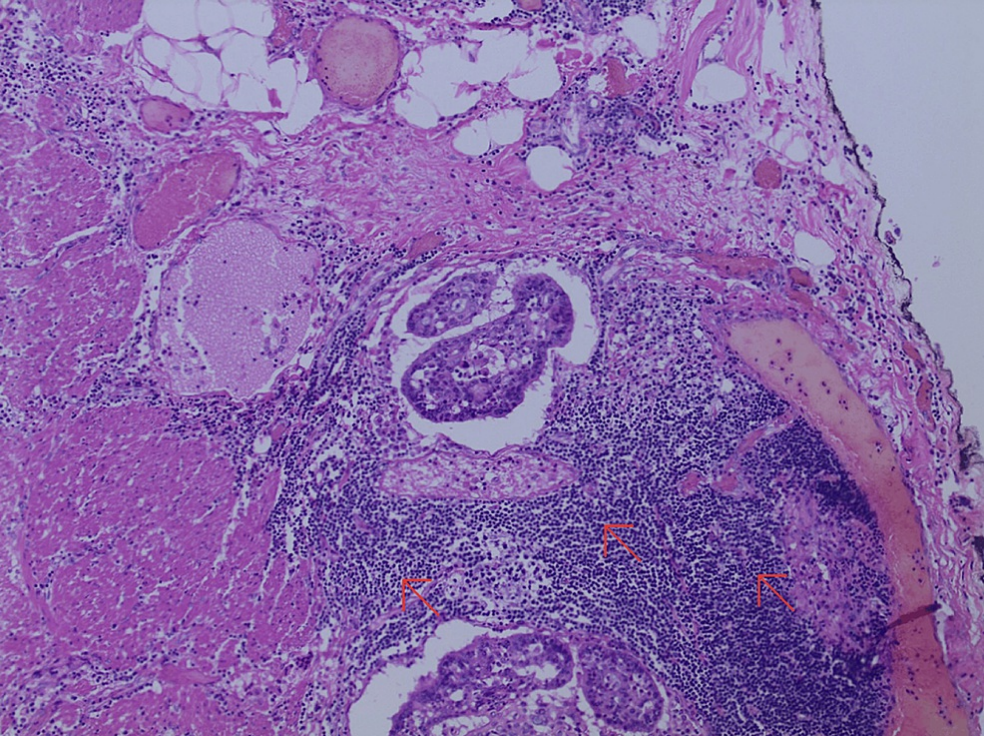
Histology slide of medullary carcinoma of the colon, which demonstrates dark uniform, round to polygonal cells (red arrows) with amphophilic cytoplasm and prominent nucleoli with numerous mitotic figures

**Figure 4:**
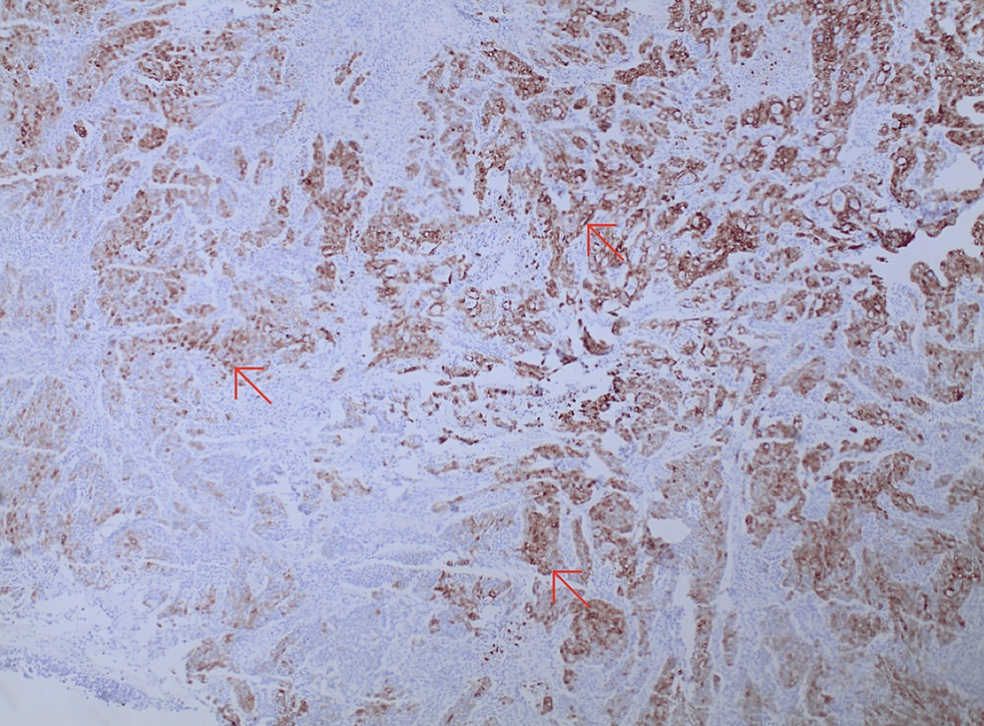
Histology slide of medullary carcinoma of the colon staining with hematoxylin and eosin positive for CK7 (red arrows)

In addition, the patient’s pathology report of the colon revealed poorly differentiated carcinoma (5 cm x 4.2 cm x 0.6 cm), consistent with MC of the colon. The malignancy infiltrated the muscular layer and focal extension into the pericolic adipose tissue, making it stage IIA (T3N0M0). The tumor was positive for lymph vascular permeation and negative for perineural invasion. Surgically resected margins were negative for tumor including 26 (0/26) mesocolon lymph nodes taken for biopsy. The cellular morphology demonstrated uniform, round to polygonal cells with amphophilic cytoplasm and prominent nucleoli with numerous mitotic figures (Figure 3). The cells stained positive for CK7 and negative for CK20, CDX2, and synaptophysin immunostains (Figure 4).

**Figure 5:**
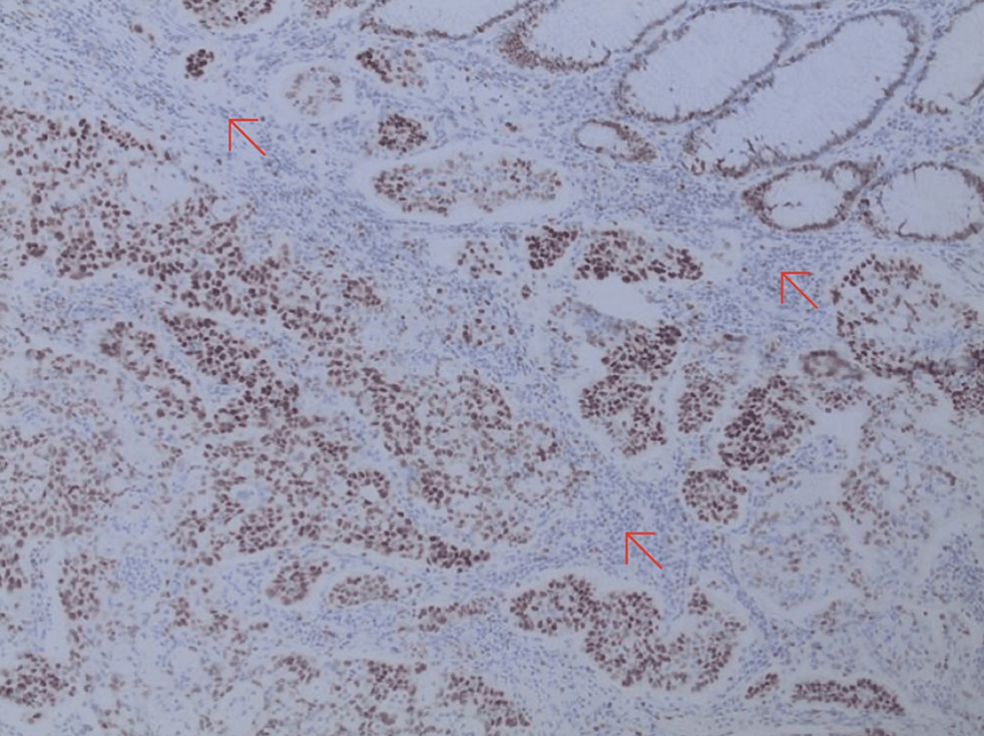
Histology slide of medullary carcinoma of the colon, which demonstrates light blue cells lacking expression of MLH-1 and PMS2 (red arrows)

Calretinin was focally and weakly positive. Mismatch repair (MMR) proteins' immunohistochemistry revealed loss of MLH-1 and PMSH2 nuclear expression with low microsatellite instabilities (Figure 5).

## Discussion

MC of the colon is recognized as a subtype of adenocarcinoma. Unlike the typical glandular cells in PDC, MC is composed of sheets of malignant cells with vesicular nuclei and the absence of MLH-1 [6]. Studies conducted by Boland et al. on the treatment of CRC with adjuvant chemotherapy demonstrated survival benefits for patients with stage III and high-risk stage II colon cancer [6]. Stage-based treatment of colon cancer over the last decade has been subjected to scrutiny over the benefits of adjuvant chemotherapy according to staging. The National Comprehensive Cancer Network (NCCN) guidelines for the treatment of colon cancer recommend stage I and low-risk stage II disease should be treated with surgery alone [7], while a patient with stage III or high-risk stage II colon cancer should be treated with surgical resection and ACT. The NCCN has strong evidence in favor of treating stage III CRC with adjuvant chemotherapy like oxaliplatin, fluorouracil, levamisole, and leucovorin, which are associated with improvement in survival outcomes [5,7-9].

In contrast, the treatment approach of stage II colon cancer with high risk has been subjected to ongoing controversy. High-risk stage II colon cancer is defined by tumor depth, histologic grade, margin status, and number of nodes biopsied [7]. While some studies have shown an overall increased survival rate in addition to adjuvant chemotherapy with surgery, other studies have not. Hence, while the NCCN guidelines recommend adjuvant chemotherapy for patients with high-risk stage II, many patients will not receive adjuvant chemotherapy due to uncertainties in treatment benefits. In stage II (node negative) disease, the addition of adjuvant chemotherapy is influenced by the tumoral deficiency of DNA mismatch repair enzyme status and high levels of microsatellite instabilities (dMMR/MSI-H) [10]. However, the determination of high-risk colon cancer guidelines proposed by the American Society of Clinical Oncology (ASCO), European Society for Medical Oncology (ESMO), and NCCN do not recognize MC of the colon as a high-risk disease. Therefore, patients with MC are not considered favorable candidates for adjuvant chemotherapy. Knowingly, MC is a consequence of MMR deficiency and a high level of microsatellite instabilities, which is associated with an overall better prognosis [10].

Regarding early-stage colon cancer, the definition of high-risk varies among experts. According to the College of American Pathologists (CAP) guidelines developed in early 2000, the disease is labeled as high risk if T4 disease, nodal status, lymphovascular invasion (LVI), a high pre-operational level of carcinoembryonic antigen (CEA), and positive surgical margins are present. These guidelines set forth by CAP have not been updated since 2000. Similarly, guidelines made by ASCO, ESMO, and NCCN have similar criteria with the addition of perforation, clinical obstruction, perineural invasion, and high tumor budding score (BD3, >10 buds) [10]. These guidelines fail to recognize MC as a high-risk feature. Interestingly, the pathology report from the patient determined that her tumor had lymphovascular invasion. Based on this finding and her rare form of colon cancer, it could be argued that she should have received an assessment of high risk and deemed qualified for adjuvant chemotherapy solely based on her tumor being stage II with lymphovascular invasion. As her diagnosis is MC of the colon, it should have collectively served as an additive reason for her high-risk status.

Based on the evidence presented regarding the MC of the colon, it would be wise to suggest the addition of stage II MC to the high-risk category for recommendations of adjuvant chemotherapy. Patients with stage IIA MC with MMR protein deficiency, like the patient discussed in this case, should be considered a prime candidate for adjuvant chemotherapy. Studies have shown that approximately 5% to 15 % of CRC have sporadic or inherited deficiency in MMR proteins (Lynch syndrome) [10]. In stage II disease, MMR status influences the recommendation for adjuvant chemotherapy. Patients with a deficiency in MMR proteins have a 50% lower risk of recurrence and, therefore, a higher survival rate after surgery [10]. While most studies confirm that MMR protein-deficient colon cancer is resistant to fluorouracil-based adjuvant chemotherapy, it remains sensitive to oxaliplatin [10].

## Conclusions

MC of the colon is a rare malignancy with distinct histology and immunochemistry. While this disease is associated with Lynch syndrome, it carries a relatively good prognosis if diagnosed in the early stages. In particular, stage II MC of the colon is sensitive to adjuvant chemotherapy but often does not qualify for treatment based on the high-risk guidelines. Treatment with chemotherapeutic drugs such as oxaliplatin and fluorouracil has proven to be an effective addition to prevent recurrence. In the future revision of colon cancer high-risk guidelines, the ASCO, the ESMO, and the NCCN can all recognize the importance of adding MC to the guidelines. Based on the evidence presented, it would be an argumentative proposal to add MC to the high-risk criteria so that patients with MC can be considered eligible candidates for chemotherapy. While the approach for tumors with MMR deficiency is uncertain, perhaps revised high-risk guidelines can recommend adjuvant chemotherapy.
